# Magnetically Recoverable TiO_2_/SiO_2_/γ-Fe_2_O_3_/rGO Composite with Significantly Enhanced UV-Visible Light Photocatalytic Activity

**DOI:** 10.3390/molecules25132996

**Published:** 2020-06-30

**Authors:** Reyhaneh Kaveh, Maryam Mokhtarifar, Mojtaba Bagherzadeh, Andrea Lucotti, Maria Vittoria Diamanti, MariaPia Pedeferri

**Affiliations:** 1 Department of Chemistry, Materials and Chemical Engineering, Politecnico di Milano, 20124 Milano, Italy; reyhanehkaveh@yahoo.com (R.K.); maryam.mokhtarifar@polimi.it (M.M.); andrea.lucotti@polimi.it (A.L.); mariapia.pedeferri@polimi.it (M.P.); 2 Department of Chemistry, Sharif University of Technology, Tehran 145888-9694, Iran; bagherzadeh@sharif.edu

**Keywords:** TiO_2_, γ-Fe_2_O_3_, SiO_2_, reduced graphene oxide, photocatalysis, methylene blue

## Abstract

In this paper, we report the preparation of a new composite (TiO_2_/SiO_2_/γ-Fe_2_O_3_/rGO) with a high photocatalytic efficiency. The properties of the composite were examined by different analyses, including X-ray diffraction (XRD), field emission scanning electron microscope (FE-SEM), photoluminescence (PL), UV-Visible light diffuse reflectance spectroscopy, Fourier transform infrared spectroscopy (FTIR), Raman, vibrating-sample magnetometer (VSM), and nitrogen gas physisorption (BET) studies. The photocatalytic efficiency of the proposed composite was evaluated by the degradation of methylene blue under UV and visible light, and the results were compared with titanium dioxide (TiO_2_), where degradation increased from 30% to 84% and 4% to 66% under UV and visible light, respectively. The significant increase in photocatalytic activity may be explained by the higher adsorption of dye on the surface of the composite and the higher separation and transfer of charge carriers, which in turn promote active sites and photocatalytic efficiency.

## 1. Introduction

Over the last decade, advanced oxidation processes (AOPs) have been productively applied in order to decrease a wide variety of recalcitrant and environmentally toxic products. Among AOPs, the photodegradation of contaminants in wastewater has found considerable interest [1]. Photocatalysts generate powerful reactive radicals, such as the hydroxyl radical (^•^OH), which react with the contaminants in wastewater, inducing their degradation [2,3].

Titanium dioxide (TiO_2_) is considered one of the most effective photocatalytic materials for environmental remediation due to its physicochemical properties, such as high thermal and chemical stability, excellent electronic properties, low cost, availability and low toxicity. However, because of its high intrinsic bandgap, TiO_2_ can be activated under UV irradiation, which is expensive and hazardous to provide. On the other hand, the photocatalytic efficiency of TiO_2_ is limited by the rapid recombination of photogenerated electrons (e^−^) and hole (h^+^) pairs [4,5,6]. In order to increase the photocatalytic activity of TiO_2_, many efforts, including doping with non-metal or metal elements [7,8], surface modification [9,10,11], and coupling with other semiconductors [12,13,14], have been devoted. One method is to improve the morphology and surface hydroxyls of TiO_2_ by adding SiO_2_, which is one of the best mesoporous support materials for improving the surface area of TiO_2_ [15,16]. Another method is to decrease the recombination rate of the photoinduced e^−^/h^+^ pairs by coupling TiO_2_ with narrow band gap semiconductors like Ag_3_PO_4_/TiO_2_ [17], SnS_2_/TiO_2_ [18], and CdS/TiO_2_ [19] composite catalysts.

Maghemite (γ-Fe_2_O_3_) is an outstanding metal-oxide semiconductor with a narrow band gap (~2.2 eV), which possesses better light harvesting and charge transport properties. However, the photocatalytic activity of γ-Fe_2_O_3_ is low because of the photocorrosion and high recombination rate of photoinduced e^−^/h^+^ pairs. The presence of γ-Fe_2_O_3_ in TiO_2_ composites can favor charge separation and improves the photocatalytic activity of the heterostructure dramatically. In addition, the presence of magnetic γ-Fe_2_O_3_ nanoparticles in TiO_2_ composites can overcome the difficulty of separating and recovering the prepared photocatalysts from the solution system [20,21,22,23].

Reduced graphene oxide (rGO) is considered a very promising material because of its superior charge separation ability between the intrinsic delocalized π–π electron. The rGO sheets could promote the transport of electrons between the organic pollutant molecules and composite photocatalysts. Due to the high surface area of rGO sheets, they can be applied as an excellent supporting material. Additionally, sheets of rGO remarkably enhance the surface area of the system and they can enhance the number of surface-active sites [24,25,26].

For instance, Phanichphant et al. reported the formation of BiVO_4_ via the method of co-precipitation. Synthesized BiVO_4_ nanoparticles were deposited on the sheets of rGO to prepare BiVO_4_/rGO by the hydrothermal method. The obtained results demonstrated that the BiVO_4_/rGO composite showed higher photocatalytic activity compared to neat BiVO_4_, with the photodegradation efficiency remaining stable up to multiple cycles. This improvement could be related to the high surface area available to adsorb more molecules of methylene blue (MB) and the effective charge separation of BiVO_4_ particles through the π electron in the structure of rGO [27]. Isari et al. used a simple sol-gel method to prepare a ternary nanocomposite of Fe-doped TiO_2_ that was decorated on reduced graphene oxide (Fe-doped TiO_2_/rGO), and the photocatalytic activity of the synthesized sample was investigated by the decontamination of rhodamine B under solar irradiation. According to the reported results, the value of the band gap energy of Fe-TiO_2_/rGO was remarkably lower than that of pure TiO_2_. Moreover, the quenched e^−^/h^+^ recombination rate and increased specific surface area of the prepared sample could improve the photocatalytic activity of Fe-TiO_2_/rGO under solar irradiation [28].

In this study, we developed a new magnetically separable TiO_2_/SiO_2_/γ-Fe_2_O_3_/rGO nanocomposite using the sol-gel technique. The prepared material showed excellent activity for the degradation of methylene blue under UV and visible light (Vis) irradiation. The photocatalytic efficiency was investigated, with consideration of the synergistic effects of rGO, SiO_2_, and γ-Fe_2_O_3_ nanoparticles in TiO_2_ composites.

## 2. Results and Discussion

### 2.1. Morphology

**Figure 1:**
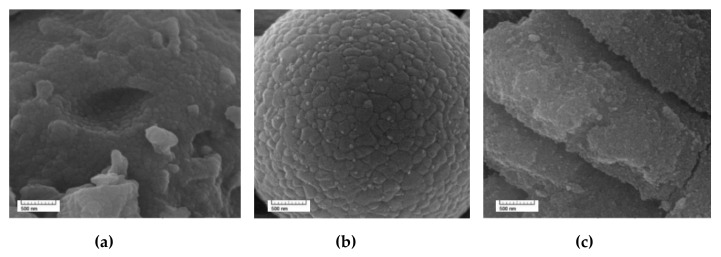
SEM image of (**a**) TiO_2_, (**b**) TS, and (**c**) TSFG.

For the morphology characterization of the synthesized samples, FE-SEM (field emission scanning electron microscope) images were taken and these are shown in Figure 1. As observed in Figure 1a, the TiO_2_ nanoparticles had a diameter between 50 and 60 nm and tended to form aggregates. In the titanium dioxide–silica (TS) nanoparticles (Figure 1b), the TiO_2_ nanoparticles had a smaller diameter and were dispersed on the SiO_2_ particles’ surfaces; hence, SiO_2_ suppresses the crystal growth of TiO_2_ nanoparticles, and it limits their aggregation. Figure 1c shows the morphology of the TiO_2_–SiO_2_–γ-Fe_2_O_3_–rGO (TSFG) sample. The particles of TiO_2_, SiO_2_, and γ-Fe_2_O_3_ were well distributed on the surface of the rGO nanosheets and served well as substrates for the homogeneous distribution of the aforementioned particles.

### 2.2. Crystal Structure of Catalyst Samples

**Figure 2:**
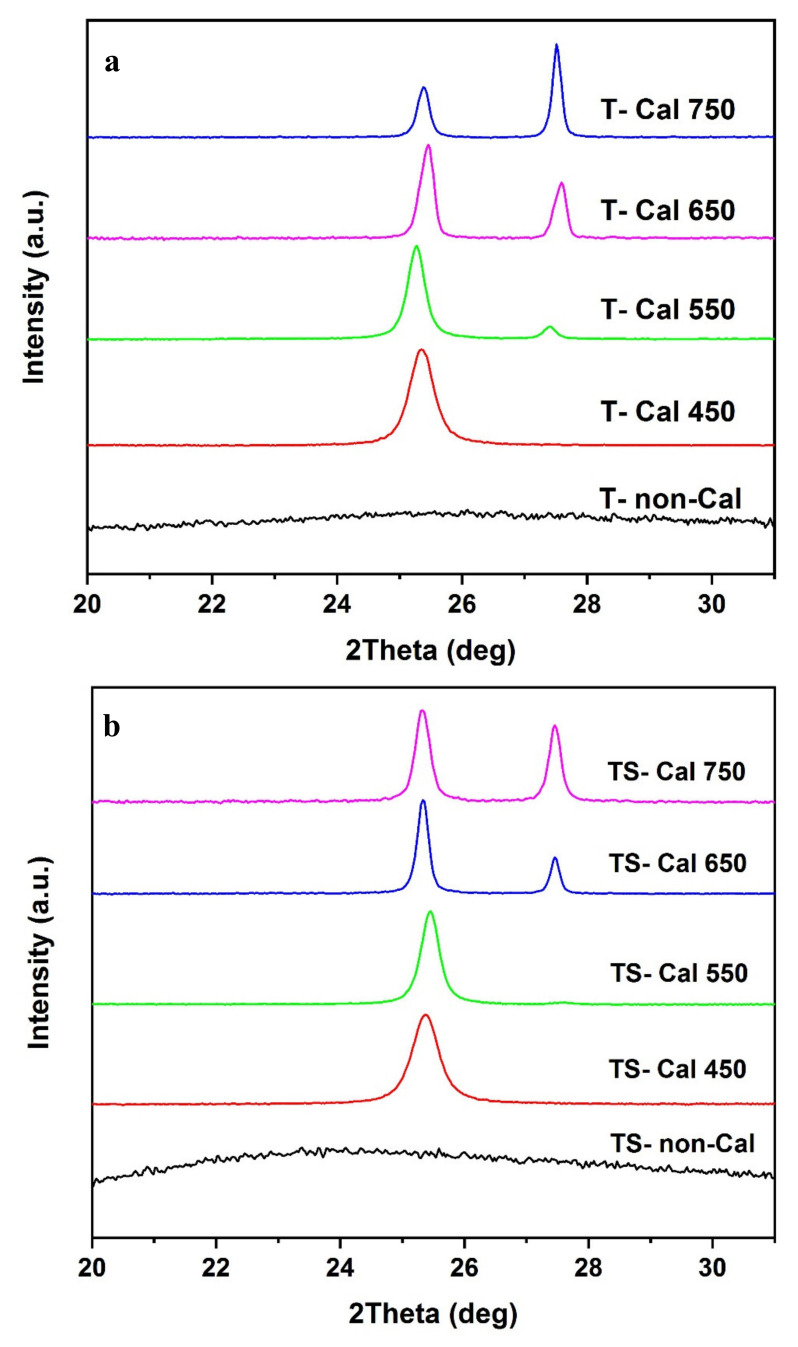
XRD patterns of (**a**) TiO_2_, and (**b**) TiO_2_/SiO_2_, after heat treatment at 450 °C to 750 °C for 120 min in a furnace.

The XRD patterns of the TiO_2_ and TS samples calcined at different temperatures are presented in Figure 2a,b, respectively. The crystalline composition is summarized in Table 1. The XRD pattern for the uncalcined TiO_2_ exhibits no peaks related to anatase or rutile, indicating that the material produced was amorphous prior to annealing. At 450 °C, anatase was detected, while the rutile phase was observed at temperatures equal to or higher than 550 °C. Increasing the calcination temperature to 650 °C resulted in a considerable improvement in the crystallinity of TiO_2_. With a calcination temperature of 750 °C, rutile became the dominant phase.

**Figure 3:**
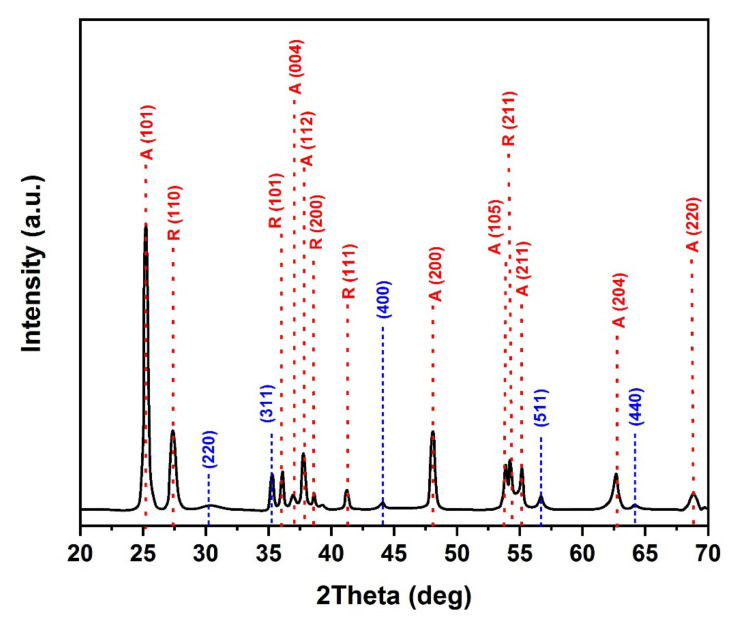
XRD pattern of TSFG after heat treatment at 650 °C.

The addition of SiO_2_ to TiO_2_ enhanced the thermal stability of the TiO_2_ crystallites, resulting in the retardation of the anatase-to-rutile phase transition (Figure 2b). This is related to the presence of SiO_2_, which prevented the nucleation of rutile: this aspect will be further analyzed with other techniques in order to understand the origin of the phenomenon [29]. According to the obtained results, TiO_2_/SiO_2_ calcinated at 650 °C may result in the best photocatalytic performance (with about 66% for anatase and 34% for rutile) if compared with the best phase composition of Degussa (P25), which has been reported by many researchers [30]. The crystallite sizes obtained by the Scherrer equation were about 29 and 24 nm for the samples of TiO_2_ and TiO_2_/SiO_2_ calcined at 650 °C, respectively; therefore, the addition of SiO_2_ to TiO_2_ hindered the growth of anatase TiO_2_ crystallites. Moreover, in addition to the peaks related to TiO_2_ anatase and rutile, the XRD pattern of the TSFG sample shows diffraction peaks corresponding to the (220), (311), (400), (511), and (440) of γ-Fe_2_O_3_ (JCPDS card 39-1356) [31], without any characteristic peaks of GO (Figure 3). Furthermore, based on Figure S1, the main peak of GO (around 11°) is not presented in rGO, proving that the GO could be successfully reduced.

### 2.3. Raman Spectroscopy

**Figure 4:**
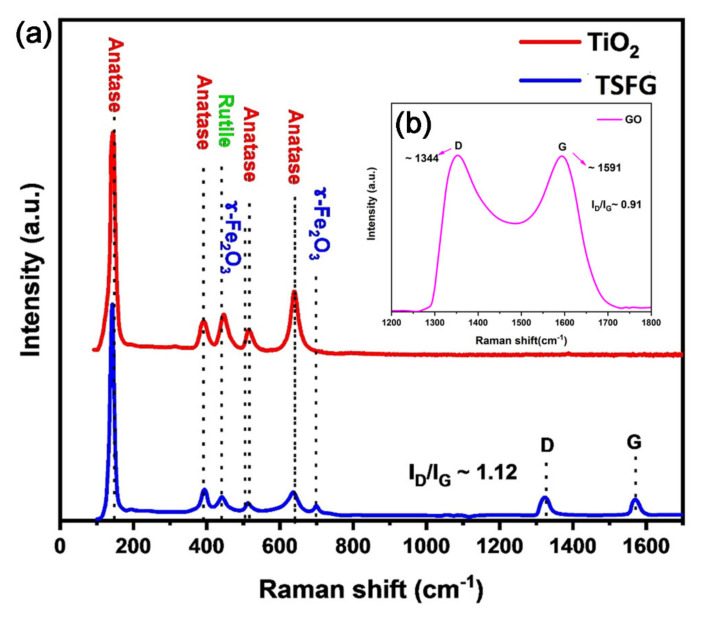
Raman spectra of (**a**) TiO_2_ and TSFG; (**b**) GO samples in the wave number range from 100 to 1800 cm^−1^.

The crystallographic structures of the prepared samples were investigated by Raman spectroscopy and the results are exhibited in Figure 4a,b. As observed in Figure 4a, the TiO_2_ anatase phase exhibited characteristic scatterings at 145, 393, and 638 cm^−1^, while the TiO_2_ rutile phase showed characteristic scatterings at 445 (E_g_) [32]. The most interesting information that can be extracted from the Raman analysis is that the position of the characteristic scatterings shifted in the TSFG sample, in comparison with the neat TiO_2_, as a consequence of the decrease in crystallite size and the lack of adjacent atoms for the surface atoms, indicating that the surface atoms were in a relaxation state [33]. Moreover, no Raman peaks corresponding to SiO_2_ can be observed; hence, either Si^4+^ is present in the substitutional positions in the TiO_2_ crystal lattice, or it is present as amorphous SiO_2_. Peaks at 513 and 700 cm^−1^ can be attributed to γ-Fe_2_O_3_, respectively [34]. Moreover, for the sample of TSFG, both the D and G bands of GO, usually located at 1323 and 1570 cm^−1^, have shifted to lower frequencies in comparison with GO (Figure 4b). This is strong evidence that GO was successfully reduced and was therefore present as rGO.

### 2.4. FTIR Spectroscopy

**Figure 5:**
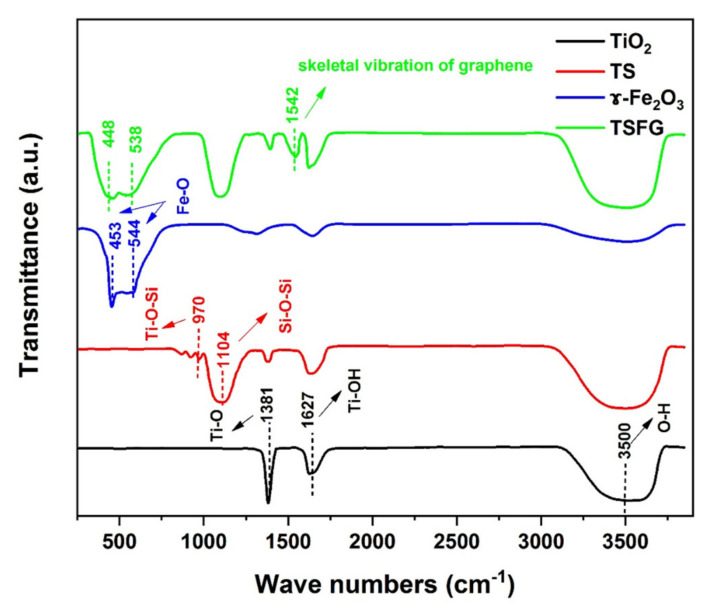
FTIR spectra of TiO_2_, TS, γ-Fe_2_O_3_, and TSFG samples for wave numbers ranging from 300 to 4000 cm^−1^.

Figure 5 shows the FTIR spectra of the TiO_2_, TS, γ-Fe_2_O_3_, and TSFG samples for wave numbers ranging from 300 to 4000 cm^−1^. TiO_2_ vividly exhibits three bands. The first broad absorption band could be observed at around 3500 cm^−1^, corresponding to the stretching vibration of the hydroxyl group O-H of TiO_2_, and the second absorption band at 1627 cm^−1^, corresponding to the bending modes of Ti-OH, indicating the surface hydroxylation of the nanoparticles. The third absorption band at 1381 cm^−1^ is attributed to Ti-O modes. The interaction between TiO_2_ and SiO_2_ in the sample of TS was exhibited in the Ti–O–Si bond (∼970 cm^−1^) [35,36]. This bond was considered to be responsible for the improvement in the thermal stability of anatase TiO_2_ that was observed by XRD, suppressing the anatase to rutile transformation.

The absorption band at 1104 cm^−1^ was attributed to the asymmetric Si–O–Si stretching vibration. Moreover, γ-Fe_2_O_3_ shows strong bands at 453 cm^−1^ and 544 cm^−1^ which are attributed to the Fe–O groups; however, TSFG exhibited vibrational bands at 448 cm^−1^ and 538 cm^−1^. This shift in the absorption band could be related to the TiO_2_/SiO_2_ surface augmentation by γ-Fe_2_O_3_ [37]. Finally, the absorption peak at 1542 cm^−1^ is related to the skeletal vibration of graphene [38,39]. Since surface hydroxyl groups are useful for photocatalytic reactions, and the TSFG sample has the largest hydroxyl peak among all samples, the samples are expected to show enhanced activity.

### 2.5. Magnetic Properties: Vibrating-Sample Magnetometer (VSM)

**Figure 6:**
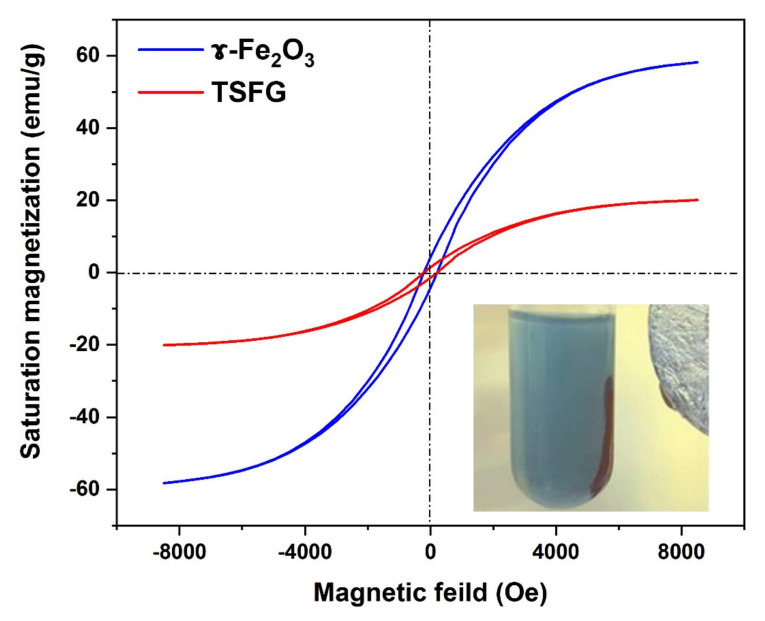
Left: the vibrating sample magnetometer curve of synthesized γ-Fe_2_O_3_ and TSFG samples. A photograph of the TSFG nanoparticles separated with a magnetic field is presented on the right.

The room temperature magnetic properties of the γ-Fe_2_O_3_ and TSFG samples are depicted in Figure 6. γ-Fe_2_O_3_ nanoparticles have a saturation magnetization (Ms) of about 58.2 emu/g, while TSFG nanocomposites have an Ms of about 20.0 emu/g. This drop is related to the presence of TiO_2_, SiO_2_, and rGO. However, 20.0 emu/g is powerful enough to respond to an external magnetic field, and it can be effectively extracted, as depicted in the same figure. Such efficient separation is required for recyclable photocatalysts.

### 2.6. Nitrogen Gas Physisorption Studies

**Figure 7:**
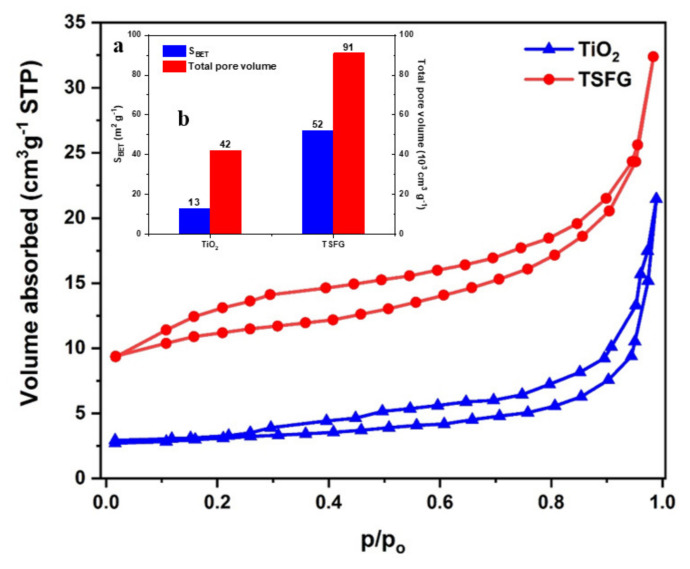
(**a**) Nitrogen adsorption–desorption isotherms; (**b**) BET surface areas and pore volumes for TiO_2_ and TSFG.

The porosity of the prepared samples was investigated by N_2_ adsorption–desorption studies. An effective photocatalyst needs a high surface area and optimum porosity. Figure 7a shows the nitrogen gas adsorption–desorption isotherms for the TiO_2_ and TSFG samples. The Brunauer–Emmett–Teller (BET) surface areas and pore volumes of these samples are given in the inset (Figure 7b). Type IV isotherms are obtained in the prepared samples, showing the presence of a mesoporous structure. The surface area of TSFG is remarkably larger than that of neat TiO_2_, owing to the incorporation of rGO sheets and SiO_2_ with netlike and porous structures [40,41]. The presence of SiO_2_ particles can retard the growth of TiO_2_ particles, and the netlike structure of SiO_2_ promoted the generation of a porous composite. The overall pore volume also increased in the TSFG composite. Enhancements in the surface area and porosity of the TSFG composite as compared to pure TiO_2_ can be beneficial, providing better interfacial contact between the surface of the catalyst and the reactants.

### 2.7. Optical Properties

**Figure 8:**
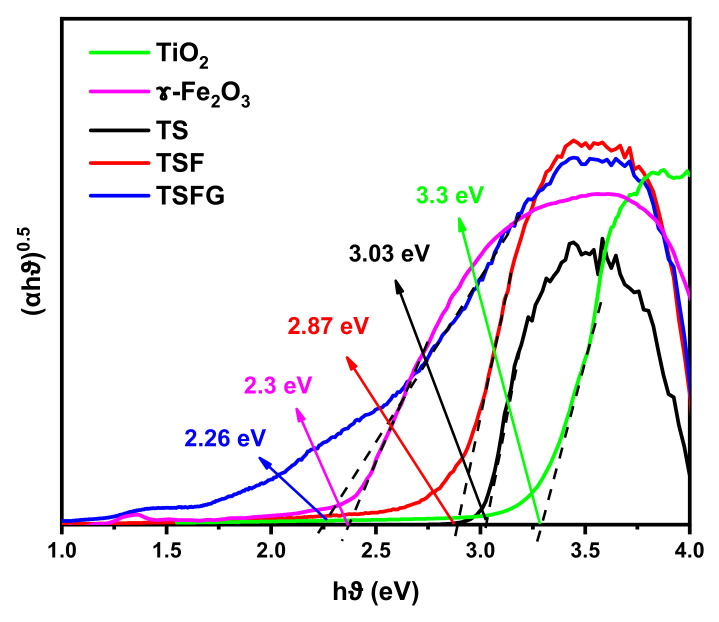
Tauc plots of (αhν)^0.5^ versus hν were employed to estimate the band gaps of the TiO_2_, γ-Fe_2_O_3,_ TS, TSF, and TSFG samples.

Figure 8 presents the direct band gap values that were determined by extrapolating the linear region of the Tauc plot of (αhυ)^0.5^ against the photon energy. The band gap energy for TiO_2_ and γ-Fe_2_O_3_ is 3.3 eV and 2.3 eV, respectively. E_g_ was calculated as 3.03 eV and 2.87 eV for TS and TSF, confirming the decrease in band gap energy produced by the addition of γ-Fe_2_O_3_. However, E_g_ is the minimum for the sample of TSFG (2.26 eV).

**Figure 9:**
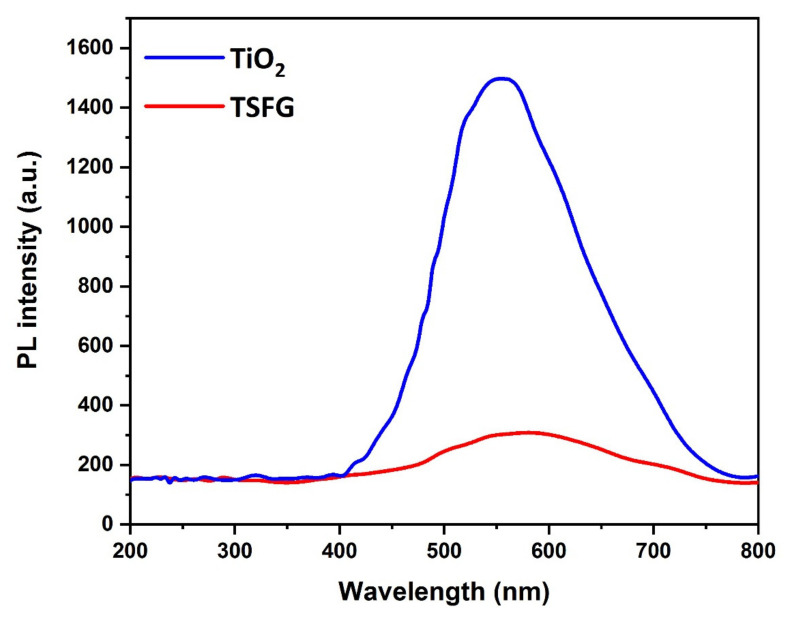
PL spectra of TiO_2_ and TSFG samples.

Photoluminescence (PL) experiments were performed to investigate the recombination probability of bare TiO_2_ and TSFG photocatalysts (Figure 9). The PL intensity of the TSFG photocatalyst was significantly lower than that of the bare TiO_2_ nanoparticles. This could be related to the lower e^−^/h^+^ recombination probability of the TSFG photocatalyst. Due to the generation of a heterojunction between TiO_2_ and γ-Fe_2_O_3_, the photogenerated electrons and holes were separated more effectively and efficiently. On the other hand, rGO speeded up the carrier mobility at the TiO_2_- γ-Fe_2_O_3_ heterojunction, increasing the photogenerated e^−^/h^+^ separation and thus possibly enhancing the photocatalytic performance.

### 2.8. Photoactivity under UV and Visible Lights

**Figure 10:**
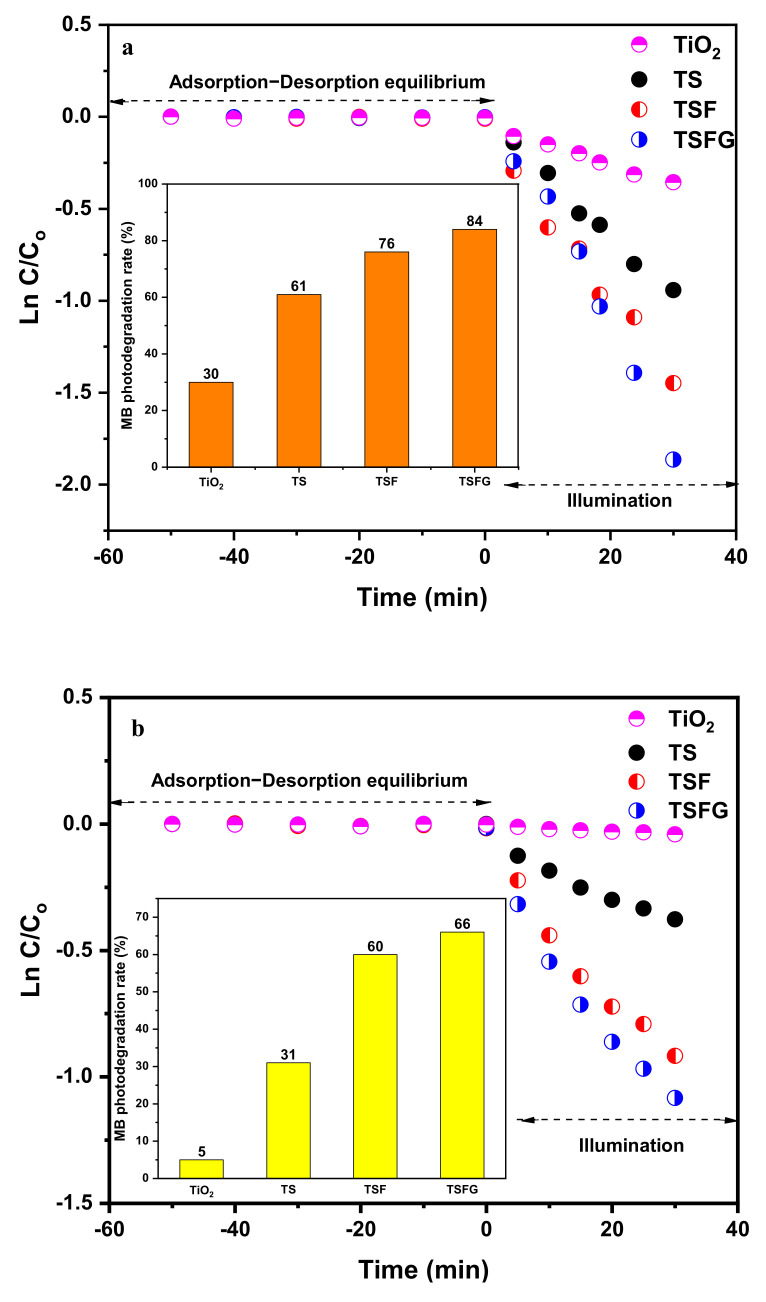
The photocatalytic activity for the degradation of methylene blue under (**a**) UV and (**b**) visible lights for TS, TSF, and TSFG systems.

The photocatalytic degradation of MB with TiO_2_, TS, TSF, and TSFG powder photocatalysts under UV and visible lights was investigated (Figure 10). After reaching the equilibrium adsorption state in the dark (50 min), it was found that the amount of MB that is adsorbed in all samples could be neglected. Additionally, in the absence of a photocatalyst, no photolysis was observed, indicating that the removal of MB only occurred through photocatalysis. As depicted in Figure 10a, pure TiO_2_ degraded only 30% of MB after 30 min under UV irradiation, while TS degraded about 61% of MB at the same condition. The samples of TSF and TSFG showed more than 76% and 84% removal under UV light irradiation, respectively. These amounts decreased to around 5%, 31%, 60, and 66% under visible light (Figure 10b). For comparison purposes, recent reports on the photodegradation of MB over different kinds of TiO_2_ composites, including Fe_2_O_3_ and graphene, are listed in Table 2.

All results displayed good linearity of ln C/C_0_ vs. time, with correlation coefficients (R^2^) higher than 0.9. Therefore, the pseudo-first-order kinetics model that was chosen was confirmed [42,43]. The kinetic constants (k) are reported in Table 3.

Some considerations are drawn here to understand the increase in photocatalytic efficiency in the different steps of composite production. Firstly, TS exhibited a higher content of anatase phase in TiO_2_ as well as an improved morphology, with smaller particle sizes and no aggregation, which made more surface sites available for reactions.

**Figure 11:**
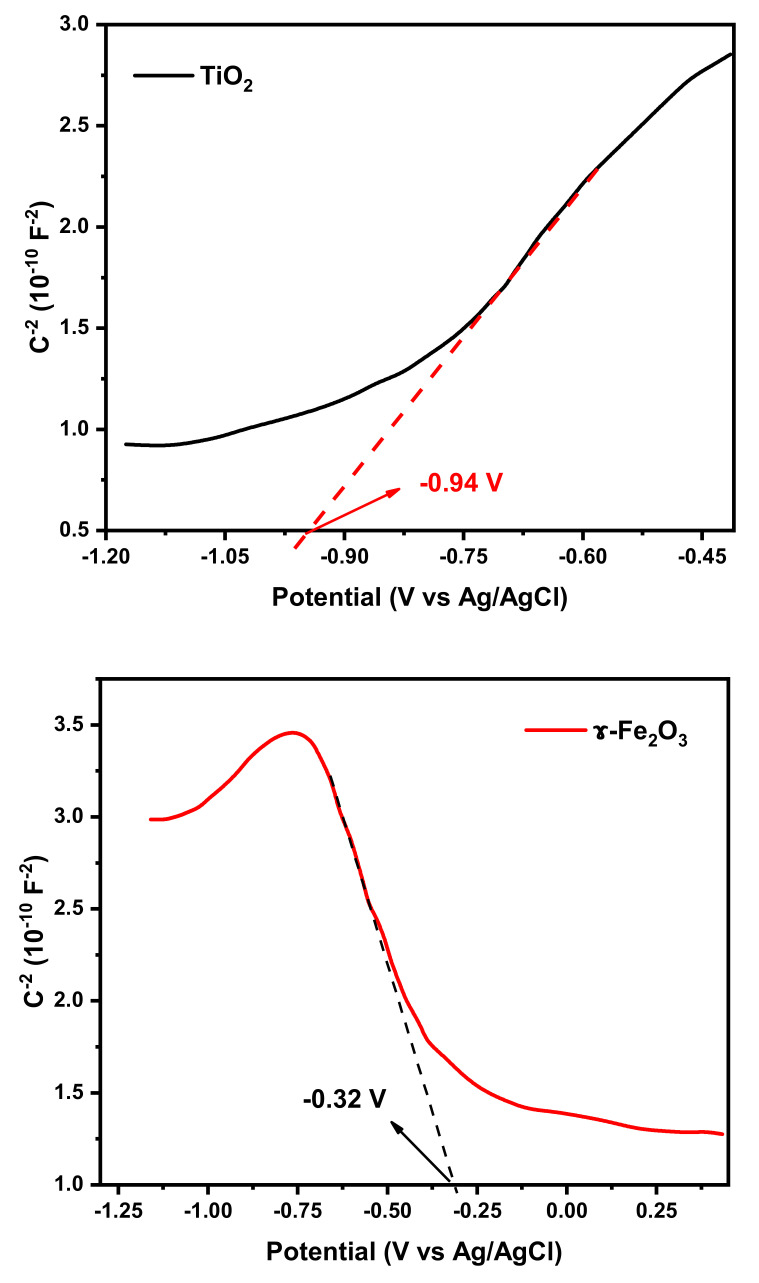
Mott–Schottky plots for TiO_2_ and γ-Fe_2_O_3_.

As observed in Figure 11, the band structures of the TiO_2_ and γ-Fe_2_O_3_ samples were estimated by Mott–Schottky plots. The positive slope of the plot confirms the extrinsic n-type conductivity of TiO_2_. On the contrary, the negative slope of the plot confirms the extrinsic p-type conductivity of γ-Fe_2_O_3_. In addition, the flat band position of the semiconductors is the interception on the X axis of the extrapolated linear flat of the Mott–Schottky plot and is close to the conduction band (CB) position of the semiconductors. Based on the obtained results, the evaluated CB positions of TiO_2_ and γ-Fe_2_O_3_ are −0.94 and −0.32 V, respectively, vs. Ag/AgCl (pH = 6.7). It should be noted that the obtained potential values are −0.34 and 0.27 V vs. NHE (pH = 0). With respect to the E_g_ of TiO_2_ (3.3 eV) and γ-Fe_2_O_3_ (2.3 eV), the valence band (VB) potentials of TiO_2_ and γ-Fe_2_O_3_ were calculated as 2.96 and 2.57 V, respectively. As mentioned, TiO_2_ is a n-type semiconductor whose Fermi energy level lies close to the CB position and γ-Fe_2_O_3_ is a p-type semiconductor whose Fermi energy level lies close to the VB position. When γ-Fe_2_O_3_ forms a heterojunction with TiO_2_, the Fermi energy levels of γ-Fe_2_O_3_ and TiO_2_ tend to rise and decrease, respectively, to reach a common value. As a result, the CB potential of γ-Fe_2_O_3_ is more negative than that of TiO_2_; hence, CB photogenerated electrons of γ-Fe_2_O_3_ could transfer to the CB of TiO_2_ and VB photogenerated holes of TiO_2_ could migrate to the VB of γ-Fe_2_O_3_ [44,45]_._ This could improve charge separation and reduce recombination. Moreover, TSFG presented a larger surface area than TiO_2_, according to the BET analyses; however, this increase is not sufficient to justify the improved efficiency observed, hence some synergistic effect is expected in the composite.

### 2.9. Effect of Reactive Species on Photodegradation Process

**Figure 12:**
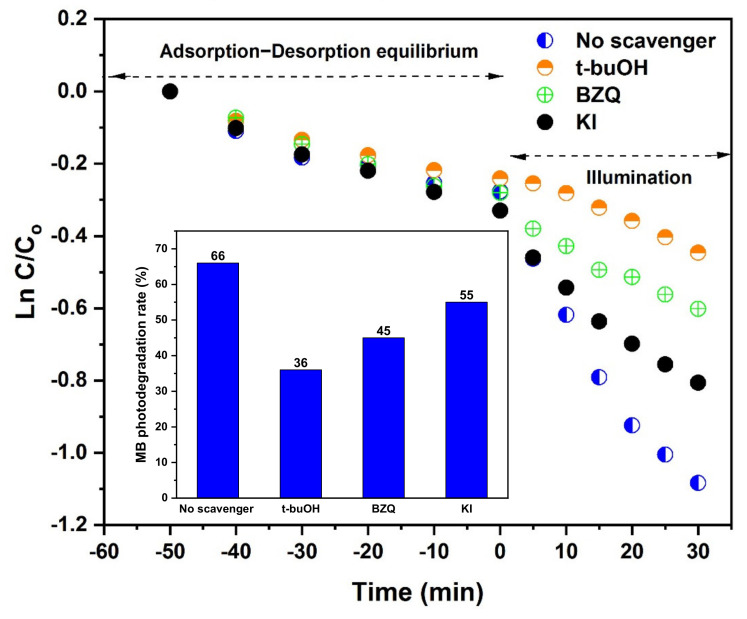
Effects of the various radical scavengers on MB photodegradation.

The types of reactive species produced in the TSFG system were determined using t-BuOH as a hydroxyl radical (^•^OH) scavenger, p-benzoquinone BZQ as a superoxide radical (O_2_^•−^) scavenger, and KI as a hole (h^+^) scavenger (Figure 12). As 1 mM t-BuOH was added, there were significant inhibitory effects on MB degradation. As 1 mM BZQ and 1 mM KI were added, there were moderate effects. These results indicated that ^•^OH was the predominant reactive species for the photodegradation of MB, while a mild reduction in MB degradation was observed by hindering oxygen superoxide and hole reactive species.

### 2.10. Photocatalytic Stability

**Figure 13:**
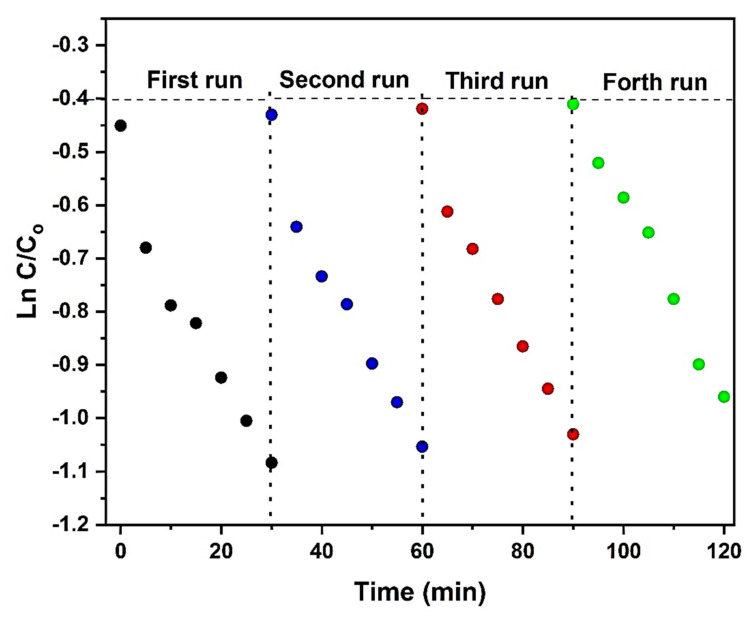
Recycle stability of the photocatalytic decomposition of MB under UV LED.

The TSFG heterojunction was recycled in four successive photocatalytic tests. At the end of the test, the used photocatalyst, separated by an external magnetic field, was washed with distilled water and dried at 80 °C for 3 h. As observed in Figure 13 and Table 4, the photocatalytic degradation of the TSFG sample shows no serious loss after four runs for the photodegradation of the MB solution. It can be clearly observed that the composite is stable during the photodegradation process and it does not suffer from either photocorrosion or poisoning in the tested environment.

### 2.11. Possible Photocatalytic Mechanism in TSFG System

The activity of the photocatalytic degradation reaction is highly dependent on a great number of parameters, such as band gap energy, adsorption of dye at the catalyst surface, particle size, crystallinity, surface area, surface hydroxylation, and e^−^/h^+^ recombination rate. For the prepared TSFG sample, the photocatalytic activity under UV and visible light illumination is related to several factors:

- The addition of SiO_2_, enhancing the hydroxyl groups and surface area of the sample and stabilizing the anatase phase
- The generation of a heterojunction between TiO_2_ and γ-Fe_2_O_3_, decreasing the rate of e^−^/h^+^ recombination
- rGO sheets have a high charge transfer ability that can increase the charge separation efficiency, also preventing recombination; they also act as nucleation sites for nanoparticle growth, allowing good distribution of nanoparticles and contrasting agglomeration.

**Figure 14:**
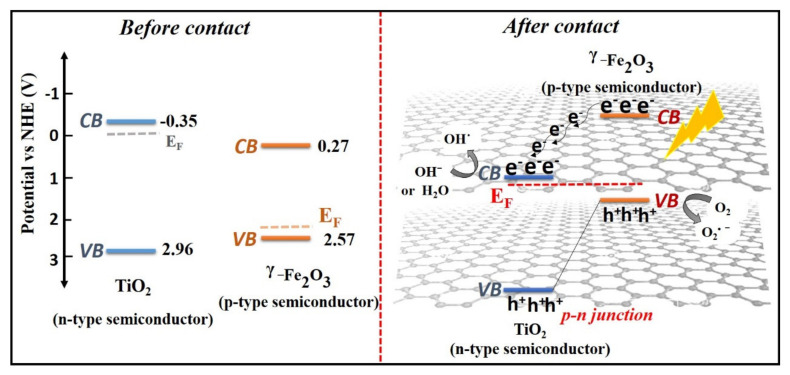
Schematic illustration of the proposed reaction mechanism for the photocatalytic degradation process in the presence of the prepared photocatalyst.

**Figure 15:**
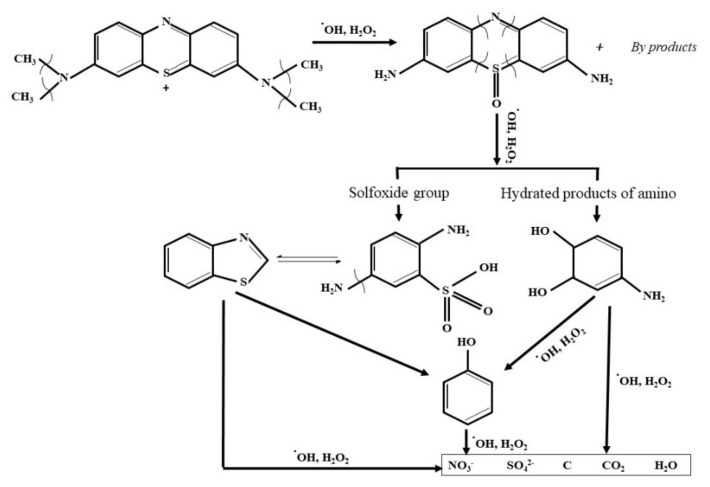
Possible degradation mechanism of MB.

Concerning visible light activity, it is negligible for pure TiO_2_ while the efficiency increases in the composite materials. Under visible light illumination, TiO_2_ cannot be excited to generate e^−^/h^+^ pairs, whereas γ-Fe_2_O_3_ can be activated to yield charge carriers. The rGO sheets can serve as the electron reservoirs to rapidly capture or shuttle the photo-induced electrons from the CB of γ-Fe_2_O_3_. Subsequently, electrons are moved to the CB of TiO_2_ by the conductive network of rGO because of the coupling interfacial contact between rGO and TiO_2_, and the photoinduced holes are accumulated in the VB of γ-Fe_2_O_3_ (Figure 14). Moreover, rGO sheets can delay the agglomeration of nanoparticles. The negative electrons in the CB of TiO_2_ react with the O_2_ dissolved in the dye solution to form O_2_^•−^. Moreover, O_2_^•−^ reacts with H^+^ to form ^•^OOH, followed by rapid decomposition to ^•^OH radicals, while the accumulated holes in the VB of γ-Fe_2_O_3_ could oxidize H_2_O and OH^−^ to form ^•^OH radicals that are involved in the photodegradation reaction of organic contaminants [52,53,54,55,56]. The photodegradation pathway of MB over the photocatalysts is schematically depicted in Figure 15 [57,58,59].

## 3. Materials and Methods

Iron chloride tetrahydrate (FeCl_2_·4H_2_O), propylene oxide, tetraethyl orthosilicate (TEOS), tetrabutyl titanate (TBT), sulfuric acid (H_2_SO_4_), graphene oxide solution (GO), sodium borohydride (NaBH_4_), ethanol (C_2_H_5_OH), and MB were purchased from Sigma–Aldrich, Milan, Italy. All reagents were used as received.

### 3.1. Preparation of γ-Fe_2_O_3_ Nanoparticles

A volume of 5 mmol of FeCl_2_·4H_2_O was dissolved in ethanol (0.3 M), and 50 mmol of propylene oxide was added to the solution, after which it was sonicated for 15 min. The obtained brown solution was stirred for 6 h and the mixture solution was boiled until powder was obtained [60].

### 3.2. Preparation of Reduced Graphene Oxide (rGO)

A volume of 6 mmol of NaBH_4_ was dissolved in diluted water (0.3 M) and then the solution was sonicated for 10 min. The aqueous solution was added dropwise to the 5 mL of homogeneous GO aqueous dispersion (2 mg/mL) while it was being stirred at 80 °C for 1 h. The suspension was centrifuged and washed several times to obtain the black powder of rGO [61].

### 3.3. Preparation of Nano Magnetic TiO_2_/SiO_2_/γ-Fe_2_O_3_/rGO Photocatalyst

A volume of 40 mmol of TEOS was added to 65% HNO_3_ and the solution was stirred for 1 h (molar ratio of TEOS and H^+^ was kept at 2.4). The product was centrifuged and dried at room temperature for 10 h. The obtained powder was dissolved in 10 mL of ethanol under sonification for 1 h, and then 400 mmol of propylene oxide and 40 mmol of TBT were added to the solution to form sol A, which was then aged for 72 h. The sol A was mixed with 0.032 g γ-Fe_2_O_3_ nanoparticles and the mixture was sonicated for 1 h. After this, 0.02 g of the obtained rGO powder was mixed with Sol A, which consisted of TiO_2_, SiO_2_, and γ-Fe_2_O_3_ nanoparticles, and the final sol was vigorously stirred for 2 h (γ-Fe_2_O_3_ and rGO amounts were optimized, see Figure S6). The sol was dried at 80 °C and then the obtained powder was calcinated for 2 h at different temperatures (Table 5). The synthesized sample was named as the TSFG photocatalyst.

## 4. Characterization

The morphologies of the prepared samples (TiO_2_, TS, and TSFG) were evaluated by field emission scanning electron microscopy (FE-SEM, TESCANMIRA ΙΙ, Brno-Kohoutovice, Czech Republic). For sample preparation, the synthesized nanoparticles were dispersed in ethanol and then a drop of the mixed solution was withdrawn and dried on an aluminum plate. X-ray diffraction (XRD) was used to investigate the crystalline structures present in the photocatalyst samples, and then the diffraction patterns were recorded on a Philips PW1830 powder diffractometer (Amsterdam, Netherlands) operating at a 40 kV voltage and a 40 mA filament current. Spectra were acquired at a scanning rate of 2.5° per min with Cu K_α1_ radiation in the 2θ range of 20–60°. According to the Debye–Scherrer equation, the TiO_2_ crystal sizes of the samples were obtained, with reference to the strongest diffraction of the anatase (110) plane. The weight fraction of anatase with respect to the whole amount of the TiO_2_ crystalline phases (anatase, rutile) was calculated according to Equation (1) [62], where *I_R_* is the intensity of the strongest rutile reflection, (110), and *I_A_* is the intensity of the strongest anatase reflection, (101).

(1) $$f_{A}=f_{A}=\frac{1}{\left(1+1.26\;\frac{\left(I_{R}\right)}{\left(I_{A}\right)}\right)}\;\%$$

Raman spectra were measured with a LABRAM HR800 (Kyoto, Japan) equipped with a Peltier cooled CCD detector. The λ = 514 nm excitation was done by an argon ion laser (Stabilite 2017, Spectra Physics (Santa Clara, CA, USA)). The laser’s radiation was filtered by an interference filter and focused on the sample by an Olympus BX41 microscope (Tokyo, Japan). A 50X Olympus objective with a 0.7 numerical aperture was utilized. The Rayleigh radiation was rejected using Notch filters for the λ = 514 nm laser line.

Optical properties were investigated by UV-Vis-NIR diffused reflectance spectra, recorded in the 220–2600 nm range with a Shimadzu UV3600 Plus spectrophotometer that was equipped with an ISR-603 integrating sphere (Kyoto, Japan), and BaSO_4_ was used as the reference material. The band gap was calculated based on the reflectance of the UV-Vis spectra after Kubelka–Munk conversion using the Tauc plot method [63]. The fourier transform infrared spectroscopy (FTIR) spectra of the prepared samples were recorded on a Bruker Tensor 27 spectrometer (Billerica, MA, USA) using a KBr pellet for sample preparation at room temperature. PL measurements were performed using CARY ECLIPSE (Santa Clara, CA, USA).

The surface area was measured using nitrogen adsorption–desorption at 77 K on Micromeritics ASAP 2020 equipment (Norcross, GA, USA), and the samples’ BET specific surface areas (SBET) were estimated. The magnetization of the prepared samples was investigated with a VSM (Lake Shore Model 7400, Westerville, United States) operating at room temperature.

The photocatalytic activity of the nanoparticles prepared here was characterized in methylene blue (MB) degradation under UV and visible irradiation. In a typical experiment, the MB solution was prepared by adding dye to deionized water (10^−5^ M) and then the synthesized photocatalysts (0.01 g) were added to the MB solution (40 mL). Prior to photodegradation under UV and visible light, the mixture was stirred for 50 min in dark conditions to reach adsorption–desorption equilibrium. After this, UV LED (500 mA, 3.8 V) and visible LED (700 mA, 3 V) were directed onto the solution. The distance between the light source and dye/catalyst solution was kept at 3 cm. The variation in the MB absorbance at given time intervals of illumination was monitored by spectrophotometry (UV-Vis spectrophotometer, Thermoscientific Spectronic 200E) at a wavelength of 668 nm, at which MB has its maximum absorbance. In separate controlled experiments, the MB solution was illuminated with UV and visible light in the absence of the prepared catalysts (photolysis experiments); this enabled the investigation of the true photocatalytic nature of the synthesized nanocomposites by discarding the component of simple photolysis. MB absorbance was then correlated with its concentration through the Beer Lambert law, with a linear proportionality between the two. Eventually, the photodegradation of MB was calculated as a function of the relative MB concentration versus the time of the reaction, C/C_o_, where C_o_ is the initial concentration of MB solution and C is the concentration at sampling time. A pseudo-first-order kinetics was hypothesized to control the rate of MB photodegradation, as observed in related scientific literature [36,64], so the slope of the curve of ln(C/C_o_) versus the time of the reaction was taken as the MB photodegradation rate constant *k*.

Mott–Schottky measurements were performed via an Autolab potentiostat/galvanostat apparatus in a three-electrode cell. Pt wire as the counter electrode, Ag/AgCl (saturated KCl) as the reference electrode, and 0.5 M Na_2_SO_4_ solution as the electrolyte were employed. The Mott–Schottky measurements were recorded at an open-circuit voltage in a frequency ranging from 0.1 Hz to 100 kHz, with an AC amplitude of 5 mV. To fabricate the working electrode, 30 mg of the synthesized photocatalyst powder and 1 mL ethanol solution were sonicated. The obtained mixture was sprayed on a 1 × 1 cm^2^ area of FTO (fluorine-doped tin oxide) glass and allowed to dry at 400 °C for 1 h in a furnace. These electrochemical tests were carried out at room temperature (25 °C).

To more deeply investigate the dominating reactive species of TSFG for MB photodegradation, a radical scavenging test was carried out. The photocatalytic degradation of MB was repeated in the presence of the prepared photocatalyst and with the addition of tert-butyl alcohol (t-BuOH) as the •OH scavenger, KI as the hole scavenger, and benzoquinone (BZQ) as the O^2•−^ scavenger.

## 5. Conclusions

A TiO_2_/SiO_2_/ γ-Fe_2_O_3_/rGO composite was synthesized from an inexpensive sol-gel method. The investigations revealed that the addition of SiO_2_ improves the morphology, crystalline structure, and surface hydroxylation of TiO_2_ nanoparticles. A suitable amount of γ-Fe_2_O_3_ in the prepared composite can decrease the recombination rate of the e^−^/h^+^ pairs and largely improve the photocatalytic activity under visible light, and its magnetic properties allow for the simple recovery of the photocatalyst nanoparticles from the effluent to be treated. The rGO sheets, as excellent electron acceptors and transporters, can also reduce recombination; moreover, in the synthesis phase, their large surface area serves as a nucleation surface for nanoparticles and improves their dispersion. On the basis of this study, the prepared TiO_2_/SiO_2_/γ-Fe_2_O_3_/rGO composite can serve as an efficient and magnetically reusable photocatalyst for the photocatalytic purification of effluents under UV and visible light illumination.

## Figures and Tables

**Table 1:** Anatase percentage in crystalline TiO_2_ (calculated by Spurr equation) and anatase particle size (calculated by Scherrer equation) for TiO_2_ and TiO_2_/SiO_2_.

	Temperature	Anatase (%)	Anatase Particle Size (nm)
**TiO_2_**	Non calcined	-	-
	450 °C	100	15
	550 °C	83	21
	650 °C	58	29
	750 °C	30	32
**TiO_2_-SiO_2_**	Non calcined	-	-
	450 °C	100	13
	550 °C	94	21
	650 °C	66	24
	750 °C	52	26

**Table 2:** Photocatalytic degradation of MB over different composite photocatalysts, including Fe_2_O_3_ and graphene, in comparison to present case.

Composite	Light Source	Degradation Time/Rate	Degradation Rate @ 40 min	Dye Concentration	Photocatalyst Amount
**Core-shell-type TiO_2_–Fe_2_O_3_** [46]	Room light	200 min/88%	40%	100 mL, 1 × 10^−5^ M	5 mg
**Fe_2_O_3_–TiO_2_** [47]	UV light	100 min/90%	35%	40 mL, 10 ppm	4 mg
**(γ-Fe_2_O_3_@SiO_2_)n@TiO_2_** [48]	UV light	80 min/80%	70%	150 mL, 25 mg/L	10 mg
**Graphene@TiO_2_** [49]	UV light Vis light	UV 200 min/88% Vis 200 min/70%	UV 70% Vis 35%	100 mL, 10 mg/L	50 mg
**Ag–TiO_2_-graphene** [50]	UV light	120 min/45%	25%	50 mL, 5 mg/L	-
**TiO_2_/graphene oxide** [51]	UV light Vis light	UV 160 min/95% Vis 160 min/65%	UV 40% Vis 35%	100 mL, 2 × 10 ^5^ M	30 mg
**Present study**	UV light Vis light	-	UV 84% Vis 66%	40 mL, 1 × 10 ^−5^ M	1 mg

**Table 3:** The kinetic constants of photocatalytic MB degradation under UV and Vis lights.

	UV		Vis	
Sample	K (min ^−1^)	R^2^	K (min ^−1^)	R^2^
**TiO_2_**	−0.011	0.98	−0.001	0.98
**TS**	−0.032	0.99	−0.012	0.95
**TSF**	−0.046	0.99	−0.029	0.97
**TSFG**	−0.062	0.98	−0.034	0.96

**Table 4:** The kinetic constants of recycle stability of the photocatalytic decomposition of MB under UV LED.

Recycle Number	K (min ^−1^)	R^2^
**1**	−0.019	0.95
**2**	−0.019	0.97
**3**	−0.019	0.98
**4**	−0.019	0.99

**Table 5:** Calcination temperature and label of prepared TiO_2_ and TiO_2_/SiO_2_.

Calcination Temperatures	TiO_2_	TiO_2_/SiO_2_
**No calcination**	T-non-Cal	TS-non-Cal
**450 °C**	T-Cal 450	TS-Cal 450
**550 °C**	T-Cal 550	TS-Cal 550
**650 °C**	T-Cal 650	TS-Cal 650
**750 °C**	T-Cal 750	TS-Cal 750

## Supplementary Materials

The following are available online, Table S1: ɤ-Fe_2_O_3_ and rGO loading for different samples, Figure S1: XRD pattern of synthesized rGO, Figure S2: Weight loss curve profiles determined by TGA and DSC for TSFG, Figure S3: Photoactivity under UV illumination of TiO_2_/SiO_2_ before and after heat treatment at 450 °C to 750 °C for 120 min in a furnace, Figure S4: The photocatalytic activity for degradation of Methylene blue under UV illumination for different loadings of TSF and TSFG systems, Figure S5: Photoactivity under Visible illumination of TiO_2_/SiO_2_ before and after heat treatment at 450 °C to 750 °C for 120 min in a furnace, Figure S6: The photocatalytic activity for degradation of Methylene blue under Visible illumination for different loadings of TSF and TSFG systems.
