# Supplementary material for: Magnetically Recoverable TiO2/SiO2/γ-Fe2O3/rGO Composite with Significantly Enhanced UV-Visible Light Photocatalytic Activity
Source: Molecules. 2020 Jun 30;25(13):2996. doi: 10.3390/molecules25132996 (PMC7412534; doi:10.3390/molecules25132996)
Supplement: Supplementary file 1 [file molecules-25-02996-s001.pdf]

Magnetically recoverable $TiO_2/SiO_2/\gamma-Fe_2O_3$/rGO composite with significantly enhanced UV-Visible light photocatalytic activity

Kaveh Reyhaneh [1,2], Mokhtarifar Maryam [1], Bagherzadeh Mojtaba [2], Lucotti Andrea [1], Diamanti Maria Vittoria [1*], and Pedeferri MariaPia [1]

[1] Dept. of Chemistry, Materials and Chemical Engineering, Politecnico di Milano, Milano, Italy

[2] Dept. of Chemistry, Sharif University of Technology, Tehran, Iran

*Corresponding author: Maria Vittoria Diamanti, Dept. of Chemistry, Materials and Chemical Engineering, Politecnico di Milano, via Mancinelli 7, 20131 Milano, Italy, email mariavittoria.diamanti@polimi.it, tel +390223993137

Due to the various amount of ɣ-Fe_2O_3 and rGO, abbreviated names are summarized in **Table S1**.

Table s1. ɣ-Fe_2O_3 and rGO loading for different samples.

ɣ-Fe_2O_3 loading (g)		rGO loading (g)	
0.016	1- TSF	0.01	1- TSFG
0.032	2- TSF	0.02	2- TSFG
0.063	3- TSF	0.03	3- TSFG

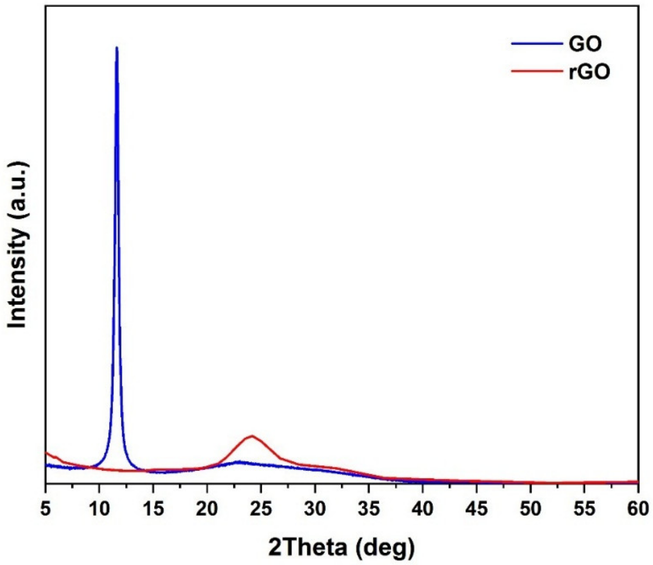

Fig. S1. XRD pattern of synthesized rGO.

Thermal stability

Thermal gravimetric analysis (TGA) and differential thermal analysis (DTA) trends of TSFG are presented in **Fig. S2** to investigate the effects of elevated temperatures upon the structure of the sample. A first mass loss is ascribed to dehydration during heating up to 150 °C [8]. The peak at 267 °C is related to the removal of residual hydroxyl groups [44]. Moreover, the peak at 409 °C could be attributed to the anatase to rutile transformation. Also, the peak at 523 °C is observed, which can be related to the oxidation of rGO [45]. Minimal weight loss was observed during heating above 650 °C. This issue cab be an evidence for the good thermal stability of the prepared sample.

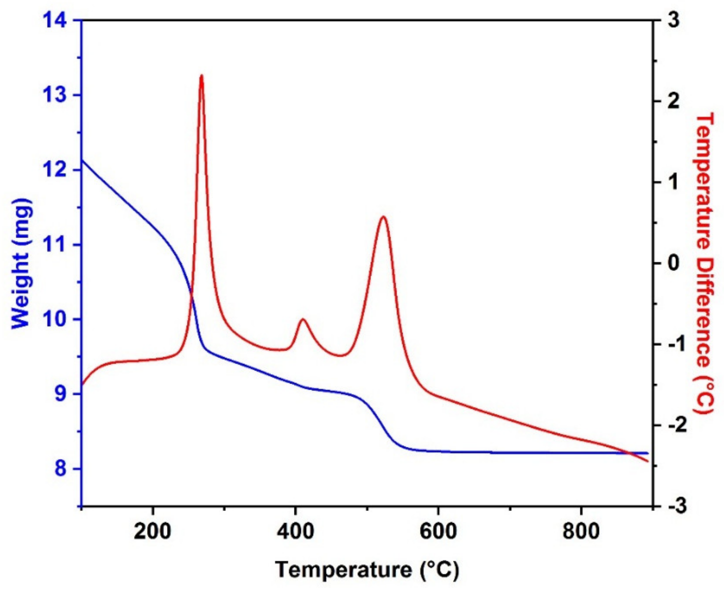

Fig. S2. Weight loss curve profiles determined by TGA and DSC for TSFG.

Photoactivity measurements

- Photoactivity under UV light

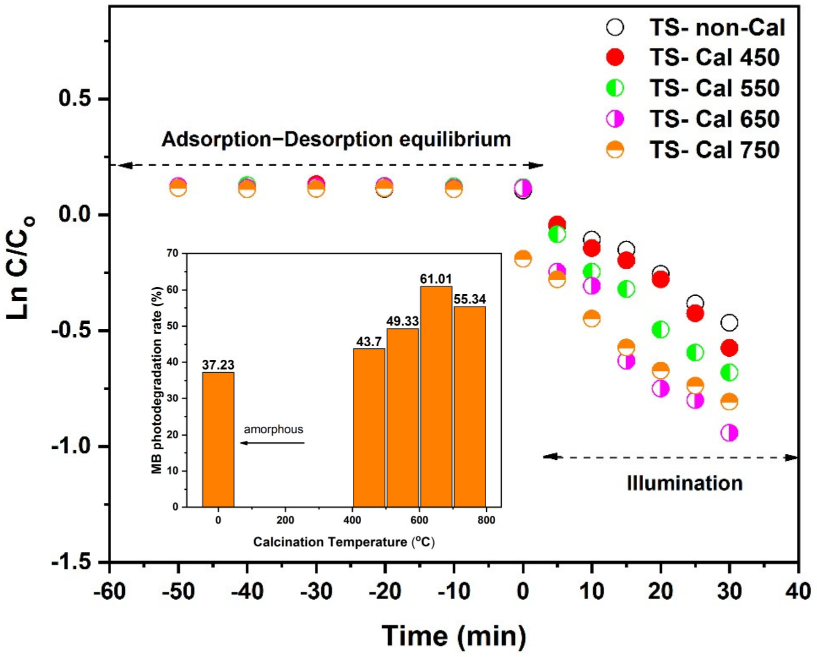

Fig. S3. Photoactivity under UV illumination of TiO_2/SiO_2 before and after heat treatment at 450 °C to 750 °C for 120 min in a furnace.

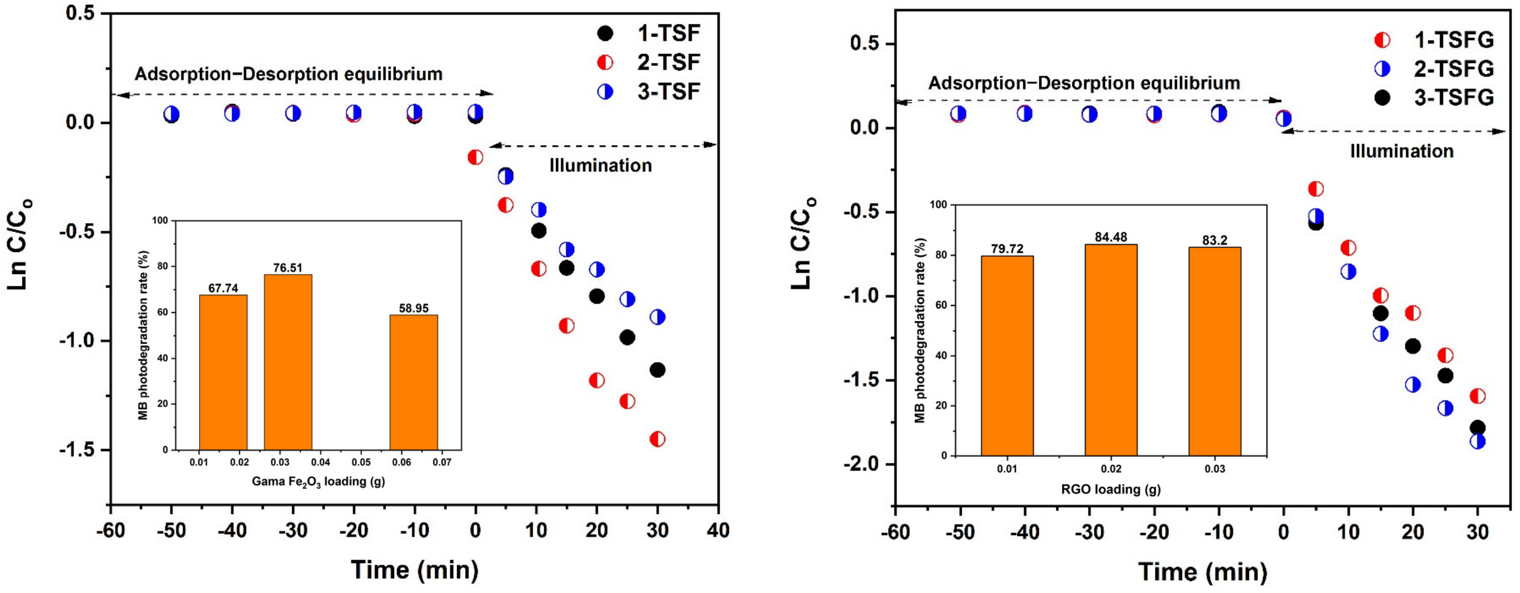

Fig. S4. The photocatalytic activity for degradation of Methylene blue under UV illumination for different loadings of TSF and TSFG systems.

- Photoactivity under Visible light

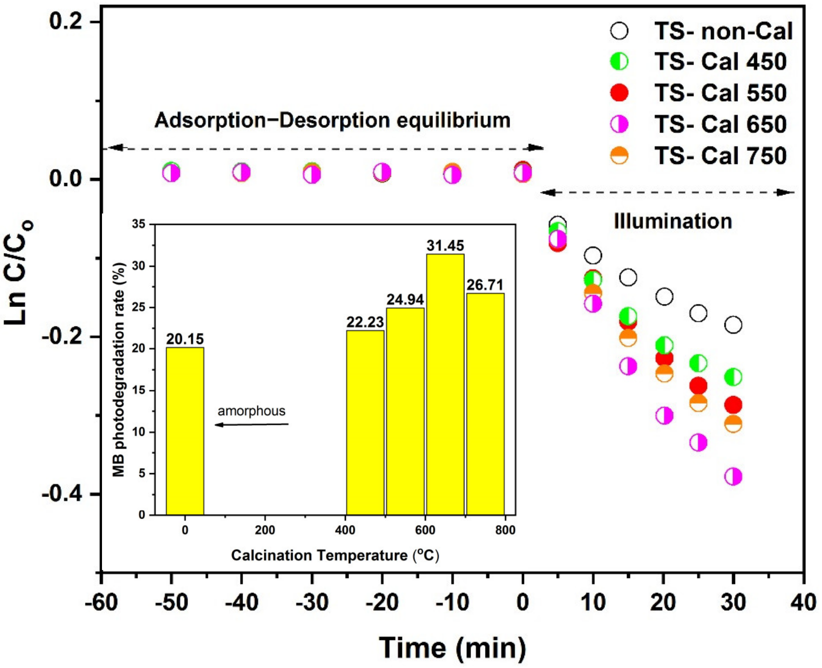

Fig. S5. Photoactivity under Visible illumination of TiO_2/SiO_2 before and after heat treatment at 450 °C to 750 °C for 120 min in a furnace.

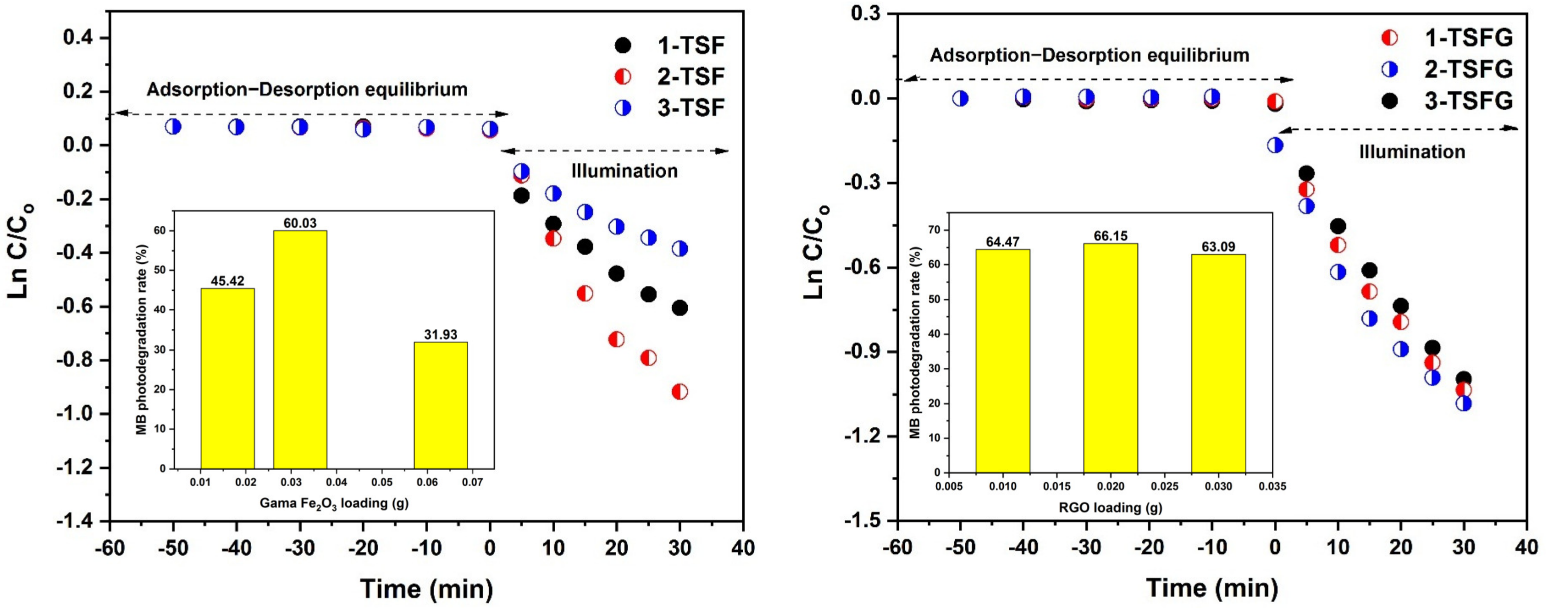

Fig. S6. The photocatalytic activity for degradation of Methylene blue under Visible illumination for different loadings of TSF and TSFG systems.
